# Continuity in the Educational Approach: Neurotech^EU^ Summer School of Quantitative Electroencephalography (QEEG) Second Edition in Cluj-Napoca

**DOI:** 10.25122/jml-2023-1027

**Published:** 2023-08

**Authors:** Livia Livinț Popa, Vlad-Florin Chelaru, Victor Dăbală, Diana Chertic, Irina Vlad, Hanna-Maria Dragoș, Dafin Mureșanu

**Affiliations:** 1 Department of Neurosciences, Iuliu Hațieganu University of Medicine and Pharmacy, Cluj-Napoca, Romania; 2 RoNeuro Institute for Neurological Research and Diagnostic, Cluj-Napoca, Romania; 3 Faculty of Medicine, Iuliu Hațieganu University of Medicine and Pharmacy, Cluj-Napoca, Romania

## INTRODUCTION

Among the neurophysiological methods of investigating the brain, electroencephalography (EEG) and quantitative electroencephalography (QEEG) are known for showing constant increases in sensitivity and specificity, and exponential advancement in clinical utility. Detection and interpretation of the human brain waves using EEG allows a deeper understanding of brain functioning. Building on this, QEEG involves the use of various algorithms leading to the transformation of brain waves into numerical data, which can be analyzed to gain insight into signal complexity, neural networks, and connections within the human brain. The technique has been applied extensively in clinical settings, including the study of neuropsychiatric disorders, human behavior, epilepsy, stroke, or traumatic brain injury.

Neurotechnology connects a wide variety of fields aiming to diminish the existing gaps in neurosciences, from medicine and psychology to assistive technologies, engineering, and artificial intelligence. Its objective is to investigate brain principles from bench to bedside, promoting a thorough understanding of cerebral processes, and the development of innovative technologies with experimental and clinical applications.

Neurotech^EU^, the European University of Brain and Technology, brings together nine prominent universities and a large variety of stakeholders covering all European regions. The goal is to facilitate collaboration among all interested stakeholders (e.g., scientific, governmental, and non-governmental organizations, patient representatives, and research institutions) thus establishing a complex European network of neuroscientific excellence focusing on neurological research and technology. The project fosters a competitive, diverse, and multilevel approach to achieve its goals.

After a successful first edition in 2022, developed as a Blended Intensive Program (BIP) in the framework of Neurotech^EU^ and Erasmus+ Programme, the University of Medicine and Pharmacy Iuliu Hațieganu (UMFIH) organized the second edition of the **QEEG Summer School (QEEGSS)** between the 17^th^ and 21^st^ of July 2023 in Cluj-Napoca, Romania.

The educational program, coordinated by Prof. Dr. Dafin Mureșanu and Dr. Livia Popa, assembled 25 junior researchers from six countries: France – Lille University (7 delegates), Germany – Bonn University (3 delegates), Cologne University (1 delegate), the Netherlands – Radboud University (1 delegate), Iceland – Reykjavik University (4 delegates), Spain – Miguel Hernández University (4 delegates), and Romania – UMFIH Cluj-Napoca (5 delegates). Most candidates had a bachelor’s degree in medicine, psychology, bioengineering, biotechnology, industrial technology, or biological sciences. However, a few medical students were also admitted to the program. Among the participants, twenty-four were affiliated with universities from the Neurotech^EU^ Alliance, while one came on demand from Cologne University.

The training adopted a basic-to-intricate approach, starting with the fundamentals of EEG and progressing to different types of QEEG analysis (frequency and time-frequency domain analysis, brain connectivity, source analysis, statistical analysis), and the association of QEEG with other technologies (e.g., transcranial magnetic stimulation (TMS) and eye tracking).

The educational event was strategically designed to maximize engagement and active learning. Each day included morning lectures followed by interactive hands-on sessions in the afternoon in two locations: the RoNeuro Institute for Neurological Research and Diagnostic and UMFIH. This format was maintained over a four-day period. On the fifth day, the participants presented their projects and were evaluated regarding their understanding of the course content, acquired skills, approach to the project, presentation skills, and originality. Subsequently, the winning team was established and awarded for their efforts.

During the program, the participants became familiar with various QEEG analysis tools, such as BrainVision Analyzer 2 and MATLAB toolboxes (e.g., Brainstorm), and engaged in team-oriented activities that promoted multidisciplinary learning and exchange of skills.

The curriculum content was structured in:

- EEG Basics;
- Theoretical Foundations of QEEG;
- QEEG Analyses;
- QEEG and Other Technologies;
- Group Projects on QEEG.

To ensure seamless implementation, the following technical resources were made available: complete equipment for EEG recording on 21-channels (Neurosoft – LIAMED), respectively 32 channels (NicoletOne™ EEG system – Natus Medical), and BrainVision Analyzer Educational Licenses offered with the courtesy of Brain Products GmbH, wherewith the preprocessing steps were accomplished. Brainstorm application for feature analysis along with the R programming language was preferred for further processes.

## EVENT SUMMARY

### First Day: Meet and Greet

The opening of the event took place at the RoNeuro Institute for Neurological Research and Diagnostic, a prestigious institution affiliated with UMFIH and a scientific partner of the Neurotech^EU^ Alliance.

**Figure 1:**
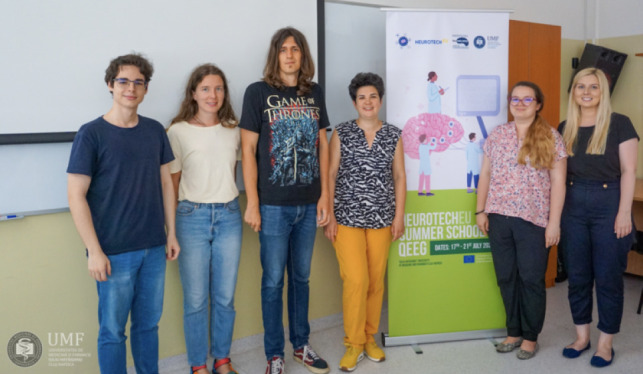
Speakers at the Neurotech^EU^ Summer School of QEEG second edition

The QEEGSS was organized under the guidance of Professor Dr. Dafin Fior Mureșanu, the Chairman of RoNeuro, Chairman of the Neuroscience Department at the UMFIH, President of the Society for the Study of Neuroprotection and Neuroplasticity (SSNN), and President of the European Federation of NeuroRehabilitation Societies (EFNR). Dr. Livia Popa, neurophysiologist and researcher, lead of the QEEG Lab at the RoNeuro Institute, emphasized in the opening remarks the enthusiasm and motivation of the local team (Figure 1) in organizing the second edition of QEEGSS under the supervision of Professor Dr. Mureșanu.

Thenceforward Dr. Popa pinpointed the primary objective of the QEEGSS, which is to deliver a comprehensive introduction to the field of QEEG for a better understanding of brain activity and inform on its wide-ranging applications in clinical practice and research. The activities covered during the summer school were then presented, namely the exploration of data collection intricacies and analyzing techniques (preprocessing and processing) and acquisition of experience in QEEG interpretation, along with the possible integration of these findings into clinical practice and more advanced topics, such as source localization and connectivity analysis. The QEEGSS students were randomly assigned to teams and worked together for the following five days on common projects fostering a spirit of cross-disciplinary learning and multidisciplinary cooperation.

The initial presentation, *The Paradigm of Brain Technology and Neuroscience* focused on the importance of collaboration between neurodisciplines grouped around the technology-dominated core. As the main scope of the European University of Brain and Technology is bridging European communities of young neuroscientists, UMFIH Cluj-Napoca plays a key role in reinforcing this position by elaborating translational and clinical research projects in several centers of excellence, such as:

- Centre for Translational Research in Neurosciences;
- Centre for Research on the Dissemination of Drug-Related Information;
- Centre of Research in Functional Genomics, Biomedicine and Translational Medicine;
- Centre for Experimental Medicine and Practical Skills;
- MedFUTURE – the Research Centre for Advanced Medicine.

The RoNeuro Institute for Neurological Research and Diagnostic represents an additional fundamental pillar of support for neuroscientific research and education materialized by the close collaboration with UMFIH and Neurotech^EU^ through its various research groups focusing on EEG, QEEG, TMS, Eye tracking, Cervical and Transcranial Sonography, besides the keen interest in Cerebrovascular Diseases, Cognitive Neurology, Neurotraumatology, Neurorehabilitation, and Neuroepidemiology.

After the introductory lecture that placed QEEG in the contemporary neurotechnological context, Dr. Diana Chertic explained the *Neurobiology of EEG Signals*. Dr. Livia Popa then offered a detailed presentation of the EEG Basic Concepts, where she illustrated the Montage selection – the arrangement of electrodes on the scalp to record EEG signals; the *Acquisition of EEG signals* – proper electrode placement, amplifying and filtering the signals to obtain accurate data; *Patterns identification* – recognizing different patterns, such as alpha, beta, theta, and delta brain oscillatory activity (waves, alpha, beta, theta, or delta rhythms) and/or epileptic discharges; *Types of activity* – alertness, resting state, drowsiness, sleep or seizure; *Artifacts* – electrode pop, electrode bridge, physiological artifacts (blink, lateral eye movement, ocular flutter, eyelid fluttering, electrocardiographic, sweat, electromyographic, glosssokinetic, photomyogenicphotogenic), as well as noise coming from electrical interference; and *Clinical applications* – epilepsy, sleep disorders, brain tumors, traumatic brain injury, stroke, encephalopathies of different etiologies.

**Figure 2:**
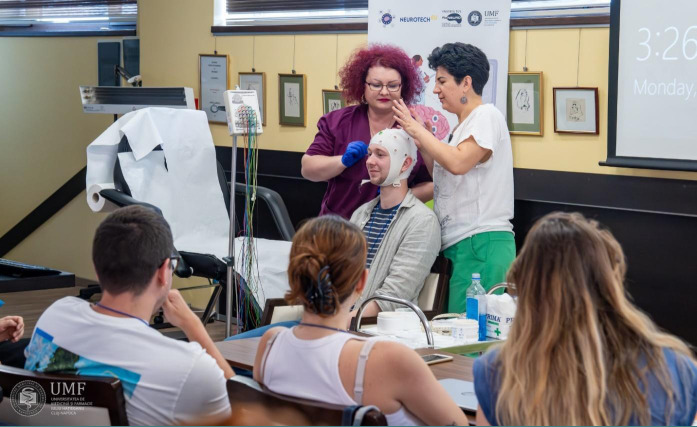
Scalp electrode placement – live EEG registration

The second part of the day focused on analyzing a live EEG recording (Figure 2). Subsequently, an initial technical setup was performed (in BrainVision Analyzer 2 and Brainstorm from MATLAB), in which most errors related to the virtual working environments and software were resolved.

### Second Day: From Standard EEG to Brain Mapping using QEEG

Dr. Livia Popa marked the start of the second day by highlighting specific methods of *Data Preprocessing, Artifact Rejection, and Reduction* (Complex demodulation, Infinite Impulse Response filters, and Band-rejection filters). Her introduction to QEEG was complemented by a brief presentation by Dr. Diana Chertic on artifact removal through the application of *Independent Component Analysis (ICA) and Principal Component Analysis (PCA)*. The theoretical part of the day was concluded by Dr. Victor Dăbală with a captivating lecture about *Frequency Domain and Time-Frequency Domain Analysis*, referring to the Fast Fourier Transform (FFT), the Dot Product, the Convolution Theorem, and the Construction of Wavelets. During the afternoon hands-on session, each student performed *QEEG Signal Preprocessing* in BrainVision Analyzer 2 and Brainstorm.

### Third Day: Brain Connectivity. Hands-on Sessions

The third day of the intensive course brought a deeper perspective into QEEG's clinical and research applicability.

The first two presentations, delivered by Dr. Hanna-Maria Dragoș, referred to the possibilities of neuronal communication through synchronicity modulation (coherence), which is included in *Frequency-Domain Connectivity*. She also covered the topics of correlation, cross-correlation, transfer entropy, mutual information, and Granger causality, which are included in *Time-Domain Connectivity*, along with explaining what a connectivity matrix represents. The theoretical discussions were complemented by clinical cases that illustrated the applications of QEEG in daily neurological practice. These cases sparked lively debates on differential diagnoses, ranging from neurodevelopmental to neurodegenerative disorders.

Dr. Diana Chertic continued the day with a presentation on *Electrophysiological Source Imaging* detection, providing several EEG source localization tools for participants.

Later on, through his presentation *Data – from Brainstorm to R*, Stud. Vlad Chelaru demonstrated a workflow for the integration of Brainstorm computations with frequentist statistical analysis software, exporting QEEG-based indexes (e.g., absolute and relative power spectral densities and correlation matrices) in a format compatible with other software packages such as R.

A workshop on *QEEG Feature Extraction*, led by Dr. Victor Dăbală and Dr. Diana Chertic concluded the day. Participants had the opportunity to transform the time series data (which were initially re-referenced, filtered, and cleared from artifacts and bad segments) into topographical maps and numerical data using BrainVision Analyzer 2 and Brainstorm algorithms.

### Fourth Day: QEEG and Other Technologies. Hands-on Sessions

The fourth day focused on blending Quantitative means of EEG analysis with complementary investigative technologies. Therefore, the presentation of Dr. Irina Vlad covered a wide array of technologies that might be utilized alongside QEEG: Functional Magnetic Resonance Imaging (fMRI), Functional Near-Infrared Spectroscopy (fNIRS), Transcranial Direct Current Stimulation (tDCS), Transcranial Alternating Current Stimulation (tACS), and Magnetoencephalography (MEG). She provided a comprehensive introduction to these technologies offering additional means of exploration when paired with QEEG. Dr. Vlad`s overview encompassed their development, functionalities, and mechanisms, in addition to the research applications. Her display, followed by two other lectures – QEEG & Transcranial Magnetic Stimulation (TMS) delivered by Dr. Anca Demea and QEEG & Eye Tracking (ET) presented by Dr. Emanuel Ștefănescu – sparked considerable interest among the audience. The majority of the participants actively engaged by asking questions and sharing their own experiences.

The second part of the day was dedicated to teamwork and project coordination. QEEGSS students had the opportunity to cooperate in 5-member groups, applying the knowledge and skills acquired during the first three days. While working on their project proposals, they utilized transversal competencies such as critical thinking, decision-making, problem-solving, and creativity.

### Fifth Day: A Celebration of Innovation and Collaboration

The fifth and final day of the QEEGSS testified to the power of collaboration, innovation, and relentless pursuit of knowledge. This day was dedicated to project presentation, as each team was randomly assembled at the beginning of the week to foster a spirit of cooperation and cross-disciplinary learning.

Throughout the week, each team worked diligently on their assignments, their efforts extending into the evenings following the day’s theoretical courses and hands-on sessions. The result was a rich tapestry of unique projects, reflecting the diverse skills and perspectives of the team members. The projects provided refreshing insights into EEG analysis, including innovative approaches to EEG signal cleaning through ICA, meticulous manual artifact-free epoch selection, and insightful comparisons of absolute and relative spectral densities.

**Figure 3:**
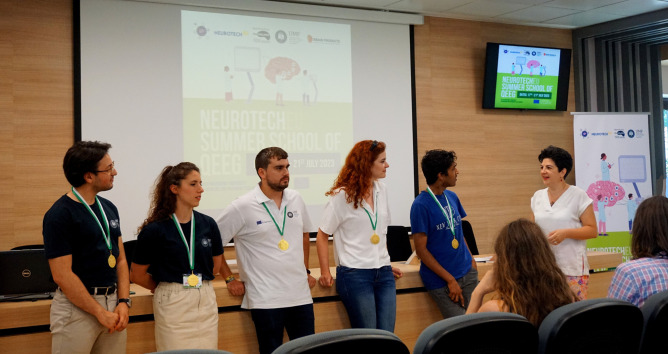
Projects assessment – The winning team (members from Lille University, Reykjavik University, Miguel Hernandez University, University of Medicine aand Pharmacy Iuliu Hațieganu, and Bonn University)

One team, demonstrating a commendable spirit of independence and technical acuity, chose to use the Python MNE package for their analysis in lieu of the BrainVision Analyzer and Brainstorm software. This decision allowed them to execute their analysis in a more controlled manner and across different operating systems, including macOS or Linux-based distributions. Another team engaged in a thoughtful critique of one literature study that focused on the same data set as the participants. They proposed several ways to mitigate the identified limitations, demonstrating a deep understanding of the subject matter and a critical approach to scientific research. This team's exceptional work earned them the winning position, and they were duly awarded for their achievement (Figure 3).

A key takeaway from the day’s presentations was the importance of standardizing analysis methods in QEEG research. Given the subjectivity of some steps, such as artifact interpretation, and the wide range of software available for analysis, one participant highlighted the need for clear and detailed descriptions of the steps used in QEEG analysis. This would ensure transparency and reproducibility, contributing to the robustness of the scientific literature in this field.

At the end of the course, the students completed an online evaluation questionnaire that assessed learning outcomes and their level of satisfaction with the entire event.

**Figure 4:**
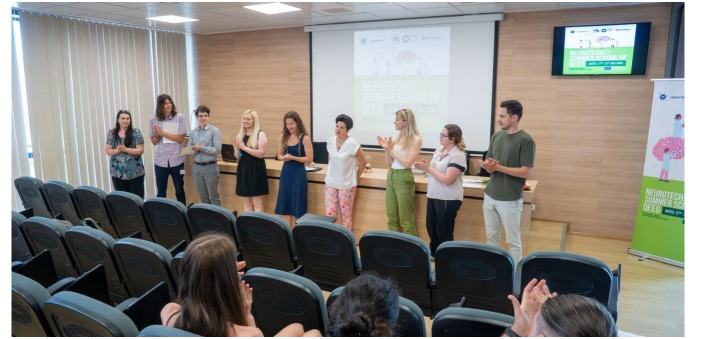
Neurotech^EU^ Summer School of QEEG second edition – Faculty at the Closing Ceremony

The closing ceremony (Figure 4) was officiated by Professor Dr. Anca Buzoianu, the Rector of UMFIH, who congratulated the participants for their dedication and enthusiasm in exploring an emerging field.

The 2023 QEEG Summer School was a journey of discovery, a celebration of collaboration, and evidence of the power of innovative thinking in advancing scientific understanding.

## IMPACT AND FUTURE DIRECTIONS

**Figure 5:**
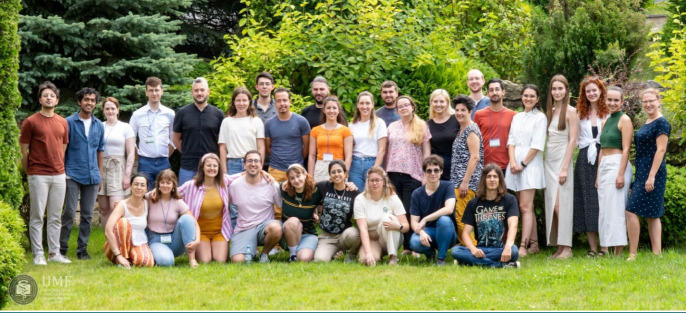
Speakers and participants at the Neurotech^EU^ Summer School of QEEG second edition

The second edition of QEEGSS (Figure 5) in the framework of Neurotech^EU^ is an educational event that confirms the strong interest of young scientists from the European consortium in exploring the intricacies of the human brain. Year after year, quantitative means of exploring neural data will gain more ground on the boundary of neuroscience and biotechnology, defining a new area in exploring brain functionality.

Through the implementation of these intensive educational programs, the scientific alliance is empowered to foster an environment of academic excellence and intellectual growth as well as be a catalyst for significant achievements in the field of neurotechnology through multidisciplinary collaborative endeavors.

